# On the Policy of Maintaining the Limits at Present Imposed by Law on the Criminal Responsibility of Madmen

**Published:** 1855-10-01

**Authors:** Fitzjames Stephen

**Affiliations:** Of the Inner Temple.


### ON THE POLICY OF MAINTAINING THE LIMITS AT PRESENT IMPOSED BY LAW ON THE CRIMINAL RESPONSIBILITY OF MADMEN.

BY FITZJAMES STEPHEN, ESQ., LL.B.,

*Of the Inner Temple.*

(Read before the "Juridical Society," 4th June, 1855.)

It is not often that we have an opportunity of laying before our readers a *strictly legal view* of the subject of Criminal Insanity from the pen of an acute and accomplished writer. We gladly avail ourselves of an opportunity of reprinting from Part I. of the papers of the "Juridical Society" Mr. Stephen's valuable essay. He has considered the subject in the spirit of a jurist, a scholar, philosopher, and a gentleman: we are bound to confess thus much, whilst we admit that we dissent from many of the propositions he has advanced. We reserve for another opportunity an expression of our critical objections to the views propounded by this writer. His essay will be read with great pleasure by all interested in this important subject.

"It is about twelve years since the public attention was called to the consideration of this subject by the murder of Mr. Drummond by Daniel M'Naughten. An impression prevailed at that period that the impunity accorded to the insane by the practice, if not by the principles, of the law, had been carried further than was consistent with the safety of society. In accordance with this policy, the House of Lords referred several questions to the Judges, in answer to which they delivered opinions which have since that time regulated the proceedings of Courts of Law in the class of cases to which they related.

"The consistency and the policy of the course adopted by the law has been lately censured with great force by some of the most eminent members of the medical profession, and in one work, of the medical merits of which I do not pretend to judge, though no one can be blind to its deep literary and philosophical interest,—I allude to the Lettsomian Lectures, lately published by Dr. Forbes Winslow,—the principles and practice of the law upon this subject are spoken of in terms which would no doubt be well deserved if the learned author had not, in my judgment at least, fallen into some confusion, not indeed as to the main doctrine of the law, but as to the course and objects of its procedure.

"Upon the question as to what the law is, there can fortunately be no doubt. I will read the question proposed by the House of Lords, and the unanimous answer of fourteen of the Judges upon the occasion to which I have referred.

*"'*Q.* What are the proper questions to be submitted to the jury, when a person alleged to be afflicted with insane delusions respecting one or more par-

* "1 Car. and Kir. 134, 135."

ticular subjects, or persons, is charged with the commission of a crime (murder, for example), and insanity is set up as a defence?'

"'In what terms ought the question to be left to the jury as to the prisoner's state of mind at the time when the act was done?'

"'*A.* The jury ought to be told, in all cases, that every man is presumed to be sane, and to possess a sufficient degree of reason to be responsible for his crimes, until the contrary is proved to their satisfaction; and that, to establish a defence on the ground of insanity, it must be clearly proved, that, at the time of the committing of the act, the party accused was labouring under such a defect of reason from disease of the mind, as not to know the nature and quality of the act he was doing, or if he did know it, that he did not know he was doing what was wrong. The mode of putting the question to the jury on these occasions has generally been, whether the accused, at the time of doing the act, knew the difference between right and wrong; which mode, though scarcely if ever leading to any mistake with the jury, is not we conceive so accurate, when put generally and in the abstract, as when put with respect to the very act with which he is charged. If the question were to be put as to the knowledge of the accused solely and exclusively with reference to the law of the land, it might tend to confound the jury, by inducing them to believe that an actual knowledge of the law of the land was essential in order to lead to a conviction; whereas the law is administered upon the principle that every one must be taken conclusively to know it, without proof that he does know it. If the accused was conscious that the act was one which he ought not to do, and if that act was at the same time contrary to the law of the land, he is punishable; and the usual course therefore has been to leave the question to the jury, whether the party accused had a sufficient degree of reason to know that he was doing an act that was wrong; and this course we think is correct, accompanied with such observations and explanations as the circumstances of each particular case may require.'

"The objections made to the law so stated by Dr. F. Winslow, Dr. Prichard, Dr. Ray, and Dr. Taylor, are, as I understand them,

"1st. That the law is guilty of inconsistency in theoretically excusing madmen from responsibility for their crimes, whilst it adopts a practical 'test of insanity' which includes them.

"2nd. That it propounds for the consideration of the jury questions involving metaphysical knowledge, couched in vague and indefinite language, and applying to a subject-matter in itself uncertain.

"In opposition to these objections, I propose, in the present paper, to explain the reasons which have led me to the conviction that the course adopted by the law is logically consistent with the rest of its proceedings; that the questions which it raises for the jury are questions proper to be submitted to their consideration, and which it is within the power of men of ordinary intelligence to solve; and that it would be impolitic to adopt any course of proceeding which would enlarge the immunities at present extended to madmen.

"I think that the unfavourable opinion entertained by physicians of the administration of the law arises principally from a misconception of the exact nature of its inquiries; for I find in several books of Medical Jurisprudence such phrases as 'plea of insanity,' and 'legal test of insanity.' I find one writer of the greatest eminence speaking of the 'rule of law, that no man is responsible, like a sane person, for an act committed by him while in a state of insanity.'* In fact, with the exception of Dr. Mayo, and perhaps of Dr. Ray, the medical writers with whose books I can profess acquaintance, seem to look upon the insanity of the prisoner as the thing to be proved, and his consciousness of right and wrong as its established legal criterion.

"I propose, then, to consider how far insanity is a defence for alleged crime.

* "Taylor, Med. Jour. 790."

and why evidence of its existence in a supposed criminal is admissible in a Court of Justice.

"I do not think that the vital distinction in this matter has been anywhere so clearly pointed out as in the separate opinion delivered by Mr. Justice *Maule*, in answer to the questions of the Judges, on the occasion to which I have referred.

"'What,' it was asked, 'are the proper questions to be submitted to the jury when a person is charged with a crime and insanity set up as a defence?'

"*Maule*, J., answered—'The questions necessarily to be submitted to the jury are those questions of fact which are raised on the record. In a criminal trial the question commonly is whether the accused be guilty or not guilty?' The jury, that is, are to inquire into nothing which is not in issue. They are impannelled to decide certain questions of fact in the negative or affirmative, and nothing is admissible in evidence unless it tends to enable them to answer these questions, or some of them. The questions are raised by prosecutor and the prisoner. The prosecutor affirming certain facts respecting the prisoner, and the prisoner either confessing or denying them, or alleging some reason why he should neither confess nor deny. Such denial, confession, or allegation, is the prisoner's plea, and if it raises a question of fact, asserted on one side and denied on the other, the jury are to decide it. First, then, madness is not a plea. The prisoner does not plead it as he would plead a pardon under the Great Seal, a former acquittal or conviction, or as he would plead to the jurisdiction. He gives it in evidence under the plea of Not Guilty. So that the very form of the proceedings implies, that, in order to entitle himself to an acquittal, the prisoner must not only show that he is mad, but that he is thereby not guilty. In more technical language his madness must be such as to enable him to traverse some one or more of the material averments of the indictment.

"When the defence rests upon proof of insanity, the fact of the killing (for the sake of simplicity I will confine my illustrations to a single crime) is generally admitted, so that the only material averments which remain to be traversed are those which charge will and malice.

* "'Malice,' or 'maliciously,' says Foster, 'mean an action flowing from a wicked and corrupt motive, a thing done malo animo malâ conscientia?' Now if a man has either no motives at all, or no power of discerning what motives are wicked and what not—in more popular language, if he cannot discern good from evil—he cannot be said to act maliciously in the legal sense of the word; and if he can show that by reason of any disease he is wholly unable, or that by reason of certain provocations defined by law, as, for example, the provocation of blows, he was temporarily unable to distinguish between good and evil, he has rebutted the presumption of malice. It is only in so far as it tends to rebut either this presumption, or that of will, that the madness of the prisoner is material. It is altogether a mistake to suppose that the point at issue is his sanity or insanity. The jury are not impannelled to try whether or not the prisoner is mad, but whether or not he is a wilful and malicious murderer. Except in so far as the first question bears upon the second it is as irrelevant as any indifferent fact, as the place of the prisoner's birth, or the names of his parents. When, therefore, in such cases the judge directs the jury to consider whether the prisoner was in a state of mind in which he was capable of distinguishing between good and evil, he is not proposing to them a test by which they may be able to determine the question whether the prisoner was mad when the act was done, he is simply directing their attention to what in the course which the inquiry has taken is the only material issue—the issue raised by the prisoner's traverse of the malice charged in the indictment.

* 'Foster, Crim. Law, 256."

"Stripped of all technicalities whatever, the transaction may be represented thus:—the prosecutor says, I charge this man with having voluntarily and wickedly killed A.B. The prisoner says, I did kill him, but not voluntarily or wickedly—for I was compelled by the involuntary action of my muscles, and exercised no volition in the matter; or, I was prevented by disease from distinguishing good from evil, and therefore could not act wickedly. The whole course of authority upon this subject agrees with the explanation of the law which I have offered.

"Lord *Hale* (1 H. P. C. 14) says, that there are two qualities which make men subject to moral government—will, and understanding. That where there is no will there is no transgression, that where there is no understanding there is no will. That therefore where there is no understanding there is no transgression. Again (p. 32), 'The trial of the incapacity of a party indicted for a capital offence is upon his plea of not guilty upon his arraignment;' and (p. 36), 'If a person during his insanity commit homicide or petit treason and recover his understanding, and being indicted and arraigned for the same pleads not guilty, he ought to be acquitted;' and then follows the reason: 'for by reason of his incapacity he cannot act felleo animo,'—feloniously.

"In Hawkins' Pleas of the Crown, in Sir W. Russell's work upon Crimes, and in the fourth volume of Blackstone's Commentaries, crimes are considered as involving two elements,—an unlawful act, and an unlawful will; and a person incapable of having an unlawful will is by these authors described as being *non compos mentis*. This phrase is by no means applied to those only whom we call mad. It is applied by all the authors I have mentioned to infants, lunatics, idiots, and drunkards—though as to these last there are various distinctions immaterial to my present purpose. In fact, in criminal law the phrase *non compos mentis* is used as an equivalent for unable to distinguish good and evil, and is so applied by Hawkins, who says, 'Those who are under a natural disability of distinguishing between good and evil, as infants under the age of discretion, idiots, and lunatics, are not punishable by any criminal jurisdiction whatever;' and in *Reg*. v. *Oxford*, 9 C. and P. 547, Lord *Denman* said, 'on the part of the defence it was contended that the prisoner at the bar was *non compos mentis;* that is, as has been said, unable to distinguish right from wrong.'

"I have met with no authority for the proposition that madness is any excuse whatever for crime, except in so far as it takes those who suffer under it out of the definitions laid down, quite irrespectively of the question of madness, for the purpose of determining what constitutes criminality. The reported cases upon this subject are as clear as the text writers.

"The law is thus laid down by Mr. J. *Tracy*, in the case of *Edward Arnold*, tried at Kingston in 1724, for shooting at Lord Onslow (16 St. Trials, 764): 'That this man shot, and that wilfully, is proved; but whether maliciously, that is the thing—that is the question. Whether this man hath the use of his reason and sense. If he was under the visitation of God, and could not distinguish between good and evil, and did not know what he did, though he committed the greatest offence, yet he could be guilty of no offence against any law whatsoever; for guilt arises from the mind, and the wicked will and intention of man, and if a man be deprived of his reason, and consequently of his intention, he cannot be guilty.'

"The same principle was acted upon in the case of *Lord Ferrers*, 19 St. Tr. 886; in the case of *Sir A. Kinloch*, 28 St. Tr. 891; and in the case of *Hanfield*, 27 St. Tr. 1282 (as I shall show more fully hereafter); in the case of *Bellingham;* and in that of *Oxford*. In the great case of *R.* v. *M'Naughten* (1 Townsend's St. Tr. 314), a different doctrine was supposed, I shall contend hereafter erroneously, to have received judicial sanction.

"It is upon these grounds that I maintain that it is not correct to charge the

Judges with having laid down a fallacious test of insanity, or with inconsistency in excusing madmen on the one hand, whilst on the other they apply a criterion bringing most madmen within the law. They have laid down no test of madness whatever. They have laid down tests of responsibility, or, more strictly speaking, have specified facts from which, when juries have found them, Judges are to infer malice; but it is no part of their duty to say how far particular diseases affect the relation of persons to such tests. 'That,' in the language of *Maule*, J., 'is a question not of law, but of physiology, and one not of that obvious nature to be inferred without proof.'

"It may, however, be contended, that however logical and self-consistent the course adopted by the law may be, the question which it proposes for the consideration of the jury is one which is not capable of being clearly asked or clearly answered. The grounds of this opinion seem to be, that the question whether a man knew that he was doing wrong, is a question which cannot be answered unless we are prepared with a definition of wrong; and that whereas the word wrong implies a deviation from some rule, it is impossible to say what that rule is.

"My answer to this objection is, that the difficulty is apparent and not real, for questions involving equal difficulties are daily submitted to juries, and it is not denied that they are rightly submitted to them.

"The question whether an act is wilful is at least as closely connected with the question of free will, as the question whether an act is malicious is connected with the question of the source and nature of moral obligations; but the question whether or not an act was wilful enters more or less into every criminal trial.

"Indeed, the specific question whether an act was malicious arises in other cases besides that of crimes committed by the insane. It is, for example, the question upon which the distinction between murder and manslaughter turns.

"The question of consent in cases of rape raises for the consideration of the jury a question at least as nicely balanced, and as nearly allied to the deepest metaphysical problems, as any connected with the freedom of the will, or the distinction between good and evil.

"Since the fact is that juries are constantly in the habit of solving questions apparently insoluble, it is obvious that the supposed difficulty is not a real one. I apprehend that a man may know that a certain act is wrong without being in any degree acquainted with any system of morals, and that others in a similar state of ignorance may infer from his conduct that he did know it. For it is one thing to know a fact, and another to know the reasons why the fact is so. The use of the words right and wrong preceded metaphysics, and extends into classes of society which know nothing of them. In fact, all metaphysics spring from language which is at first descriptive merely, and continues to be so in ordinary usage long after it has been made definite for the purposes of science. It may be extremely difficult to give a definition of wrong, but nothing is more easy than to describe some of the more glaring characteristics of the acts to which men affix that name. There may be wrong actions which are not universally disapproved of, or visited by the punishment of the law, or directly subversive of the security of society; but there can be no difficulty in saying that an action which fulfils all those conditions is a wrong action according to the common use of language, and that a man who knowingly does such an act, being aware of the circumstances surrounding it and able to judge of them, knows that he is doing wrong. Now it is only upon cases which unite in themselves such characteristics as these that juries are called upon to determine. The word 'wrong' is a word of description, and it is the peculiar province of a jury to determine whether the facts proved would in the ordinary use of language be considered as falling within that popular and descriptive language which the nature of the case constantly requires the law to use.

Thus, for example, whether a crop has been left on the ground for a reasonable time for a certain purpose; whether an offer to transfer shares was made within a reasonable time; whether an insured ship has sailed within a reasonable time—these are questions for a jury. So the questions of reasonable skill and due diligence are for the jury; and though that of probable cause is by a strange anomaly for the Judge, I think that it may be stated, as a general rule, that matter of description is a question for the jury and matter of definition for the Court.

"Upon these grounds I am of opinion, that, when a jury is asked whether a man knew that in doing a particular act he was doing wrong, they are not asked a question beyond the reach of very ordinary capacity.

"I think, therefore, that the course pursued by the law upon this subject is logical and self-consistent, and that the questions raised for the consideration of the jury are within their province.

"Such being the state of the case, would it be politic to alter it, either by erecting insanity into a plea in the strict sense of the word, or by providing that proof either of madness generally, or of the existence of some special insane delusion, should *per se* entitle the accused to a verdict of not guilty?

"The proposal to exempt madmen generally from punishment, is in effect a proposal to exempt all persons afflicted with a particular disease. It is, in fact, a proposal to enact in England the 64th article of the French Code: 'Il n'y a ni crime ni délit lorsque le prévenu était en état de démence au temps de l'action.' Now, as medical knowledge advances, the connexion of different forms of disease, which at first were not supposed to be connected, is by degrees laid open. Dr. Prichard, for example, and others, have discovered, as they say,—and I am not so presumptuous as to refuse for an instant to submit to the correctness of their observation,—that there is a sufficient analogy between those diseases of the brain which produce mania, and those which produce extreme imprudence and immorality of conduct, conjoined or not with a greater or less degree of intellectual aberration, to justify them in calling them both by the name of insanity, and describing the one as intellectual and the other as moral. So, too, a species of insanity called instinctive or impulsive, has been discovered, I believe, within the last thirty or forty years; and the disease which Lord Hale called partial insanity, and modern physicians call monomania, has been subdivided into several classes; amongst others, phonomania or homicidal madness, pyromania or arson madness, and kleptomania or theft madness. I do not mean in the least degree to deny the importance or the accuracy of these investigations. I will admit all that any physician can contend for, namely, that there are diseases of the brain, or of the nerves, or of some other part of the system, which predispose people in a greater or less degree to act in the manner described. What I contend is, that it would be inexpedient to allow the mere fact that such persons are the subjects of a disease to exempt them at once and entirely from punishment. I admit that there are some madmen whose madness destroys their responsibility. I admit that the persons whom Dr. Prichard describes as 'morally insane' are (for anything I know to the contrary) properly so designated; what I say is, that it would be most unwise to commit society at large to the principle that any disease now discovered, or hereafter to be discovered, which bears such an analogy to that kind and degree of madness which produces irresponsibility as to be called by the same name, is of itself a ground for exempting persons suffering under it from all criminal responsibility. When Coke and Hale wrote, no man was considered mad unless he were either permanently or for the time suffering under some of the most aggravated forms of the disease: since their days the subject has been studied, and numberless ramifications of the same disease have been discovered. Would it be wise to free all madmen from criminal responsibility because in former times no one was called mad unless he had ceased to be responsible?

"Such a doctrine would have a most injurious operation upon medical science; the physician would be biased in his investigations by the consciousness that he was extending criminal irresponsibility to a new class of persons by every successive discovery of a new form of disease, and he might be placed in the unpleasant dilemma of refusing on the one hand to call things by their right names, or on the other of absolving people quite capable of self-control from all responsibility for their actions. On the other hand, the administration of the law would become altogether uncertain. It would be impossible to assign any intelligible principle for the award of punishments, when by the mere extension of an old name to a new class of symptoms those whom most men would consider as the most atrocious criminals might be saved from punishment. Madness and criminal irresponsibility are, in fact, cross divisions which coincide to a very considerable degree, but by no means exactly; and unless the principles upon which the different classifications proceed be rigidly adhered to, endless confusion will be the result.

"The consequences of the doctrine that the disease, and not the results of the disease, entitle madmen to exemption from punishment are so monstrous, that when stated I can hardly imagine any one bold enough to maintain it.

"Monomaniacs are capable of acting quite rationally upon a variety of subjects, in fact, upon all subjects except those which they connect with their delusion. Now, as this disease may affect the wicked as well as the good, let us suppose that the most ordinary murderer—a man who murders for revenge or for plunder—happened also to be a monomaniac upon some subject totally foreign to his disease, ought he on proof of his monomania alone to be acquitted? I am not supposing the case of a man subject to phonomania or to pyromania, but that of a person committing murder from the commonest motives. Suppose, for example, it had been shown that the burglar who shot Mr. Hollest in resisting his lawful apprehension had at one time of his life been confined in a lunatic asylum, would that fact have had any bearing upon the question of his guilt? Yet if we are to regard the madness and not the results of it as the cause of the exemption given to people in this condition, we must say that such a man ought to have been acquitted.

"If madness were allowed *per se* to constitute irresponsibility in criminal cases, our criminal law would form a complete anomaly as compared with the rest of our system.

"Madness does not invalidate a will unless the testator was, by reason of his madness, unable to form a clear determination as to the disposition of his property, and one founded on a correct view of the facts. Madness does not necessarily invalidate a contract,* and it has been very recently determined in the Court of Criminal Appeal† that a madman is not as such an incompetent witness. In that case Lord *Campbell* remarked, 'Various authorities have been referred to which lay down the law that a person *non compos* is not an admissible witness. In what sense is the word *non compos* employed? If a person be so to such an extent as not to understand the nature of an oath he is not admissible, but a person subject to a considerable amount of insane delusion may yet be under the sanction of an oath, and capable of giving very material evidence.'

"Since all the disabilities of madmen are annexed to the consequences of their disease and not to the disease itself, and are extended no further than those consequences extend, why should not their responsibilities follow the same rule? I cannot but think that both Dr. Ray and other writers upon

* "*Monckton* v. *Cameroux*, 4 Exch. 17, (in the Exchequer Chamber); and 2 Exch. 487."

† "*Reg.* v. *Hill*, 5 Cox, 259."

this subject have fallen into a mistake in considering the law inconsistent as to the civil and criminal consequences of madness.

"'The law,' says Dr. Ray, 'invalidates a madman's contracts. Why does it hold him responsible for his crimes?' and he quotes with approval M. Georget's remark, 'Can we help wondering at those sentiments of Lord Hale, who seems to make more account of property than of life? No excuse for the unfortunate man who in a paroxysm of madness commits a criminal offence, whilst civil acts are to be invalidated when they have no relation to the insane impressions that may have influenced his conduct.'

"Facility in excusing murder is a strange proof of regard for human life; but in fact the law is perfectly consistent; it only places madmen under the same disabilities as infants, or married women, and for the same reason. Certain conditions of mind are an essential element of a contract, and certain other conditions are essential to a crime. Prove the absence of such essential conditions in either case, and you disprove the existence of the crime or of the contract.

"What has been called 'Moral Insanity' is another case of a species of madness which cannot in all cases be accepted as an excuse for crime. I find the following case in Dr. Prichard's 'Medical Jurisprudence of Insanity' (pp. 40, 41): Mr. W., aged about 40, was a corn-dealer and baker, and a man of mild and retiring disposition; steady in business, regular and domestic in his habits, highly conscientious, religious without ostentation, correct and cautious in his conversation, and kind and benevolent to all persons. His health was considered to be delicate, but he was never ill, and avoided great exertion, feeling himself not equal to it. He was a married man, and had several children, of whom he was very fond. He experienced some severe losses in his business, which weighed heavily upon his mind, and he became exceedingly depressed. He made great efforts to recover himself from his despondency, and exerted himself with the view of recovering for his family what he had unavoidably lost. He was, to a great extent, very soon rewarded for his efforts. It was was shortly afterwards observed by his friends that his increased exertions had improved his spirits, which, it was remarked, had become much more elevated than they were previous to his depression. He now began to extend his business, in which he was become more keen; he displayed more acuteness in buying and selling, and seldom trusted to others anything he could accomplish himself, and he was ever watchful of an opportunity to make purchases or to effect sales to his advantage. Those changes in his habits went on until his character of industry appeared to his friends to be over performed. His journeys became more frequent—he slept less—his temper grew hasty and irritated—this went on for about ten months; he then spent his evenings away from home, became discontented with his domestic arrangements, took to the use of stimulating liquors, formed improper connections, and at last forsook his family and his business, wandered about the country, sleeping in the open air, and subsisting by the meanest artifices. He was then confined in a madhouse.

"This person was obviously labouring under one and the same disease, in different degrees of intensity, throughout the whole of this period. Was he equally irresponsible throughout the whole time, or indeed irresponsible at all? He was at any rate during part of the time under his own command, he knew the consequences of his actions, and was capable no doubt of being acted upon by fear. Is the law, which does not recognise extreme hunger as an excuse for theft, or the deepest sense of injury as an excuse for revenge (and such feelings are as involuntary as any desires arising from disease can be), to say that because a man does not choose to resist a nervous twitching desire to do something which he knows he ought not to do, he is to stand excused for indulging himself? If that is so, we must wait to punish crime till men become criminals without motive. If in a fit of nervous irritation, caused by impatience or toothache, a man were to shoot dead some one who offended him, would he not be a murderer? If so, does the bare fact that the conduct of the man mentioned by Dr. Prichard, and of men like him, was produced by disease which ultimately might, or might not, deprive him of reason, put him beyond the pale of responsibility?

"The case of impulsive insanity furnishes almost stronger instances of the impossibility of adopting this view. It is said, that people are frequently urged by an unaccountable and irresistible impulse to kill those who are nearest and dearest to them. I do not the least deny that the fact may be so, nor that the victims of such an impulse ought not to be punished; but if the prisoner is acquitted, it must be because the impulse is irresistible, because the act is not wilful—if he is to be called insane it must be because the impulse is unaccountable; for I suppose no one would hesitate to say that a person having an unaccountable but resistible longing to kill, would be as fairly described as subject to impulsive insanity as if the impulse were irresistible. Thus the guilt turns upon the wilfulness of the act, and not upon the sanity of the prisoner.

"There may have been many instances of irresistible impulses of this kind, though I fear there is a disposition to confound them with unresisted impulses, but there have also been many in which they have been successfully resisted. Indeed, Dr. Prichard quotes several from Esquirol and Pinel. That such persons may have been suffering under the disease of insanity I can well understand; why they should be less responsible than people exposed to any other temptation I do not understand. The totally unreasonable and unaccountable wish to commit murder and suicide may range from a mere passing and momentary fancy up to an uncontrollable passion. Now since all these are but different degrees of the same disease, if it is the disease that makes the irresponsibility, acts done at the suggestion of any the least of these impulses ought not to be punished. If it is their irresistible character that excuses them, then there are cases in which madmen ought to be punished.

"Many persons who would not go the length of saying that no madmen ought under any circumstances to be punished by law, nevertheless maintain that persons subject to insane delusions should not be punished for acts done in consequence of those delusions, and complain of the harshness of the law in requiring anything further in order to justify a prisoner's acquittal than proof of the fact that he labours under an insane delusion. Thus, Dr. Prichard quotes with approval M. Georget's opinion, that 'Partial Insanity, or Monomania, excludes the idea of criminality and responsibility, and takes away from the affected person all responsibility for his actions, whatever may be the nature and extent of the illusions under which he may labour;' but with respect to what he has called moral insanity, he (Dr. Prichard) expresses some doubt. And Dr. Ray argues to the same effect, upon the following grounds:*

"1st. Amid the chaos of the sentiments and passions produced by moral mania, the power of the intellect must necessarily suffer, and instead of accurately weighing and examining the suggestions of the moral powers, it is influenced by motives which may be rational enough, but which would never have been adopted in a perfectly healthy state.

"2nd. Because the real character of his acts being misconceived, he does not associate them with their ordinary moral relations.

"3rd. Because no fear of punishment restrains him from committing criminal acts, for he is totally unconscious of violating any penal laws.

"Dr. Prichard and Dr. Taylor have carried this doctrine a step further, by

* "Ray, Med. Jur. Insan., pp. 234, 235. Dr. Ray's arguments point rather at moral insanity than at monomania, but he seems to consider that the same reasons apply to both cases."

proving that the connexion which subsists in the minds of madmen between their delusions and their actions is so arbitrary and illogical, that if it is once proved that a man has a delusion, it is impossible to say how far any crime that he may commit may or may not be connected with it.

"Thus Dr. Taylor mentions a case of a man who had some insane delusion respecting windmills, and passed his whole time in watching them. His friends removed him to a place where there were no windmills, in hopes that the fancy would wear out. He shortly after enticed a child into a wood, and mangled it frightfully in attempting to murder it. The connexion between the murder and the delusion was, that the madman thought that he might perhaps, as a punishment, be taken to some place where there were windmills.

"From this and similar instances it would appear, that, if we are to admit this doctrine at all, we must admit it to the extent of allowing all persons to go unpunished who are afflicted by any insane delusion. I will proceed to consider that proposal.

"The arguments in favour of it seem to me to be very fairly represented by Dr. Ray, but they are liable to the objection, that they prove, not that there is some peculiar reason for the exemption from punishment of persons under delusions, but that as the law now stands the existence of insane delusions will generally entitle a man to an acquittal, because it has a strong tendency to show either that he does not know what he is doing, or does not know that it is wrong.

"The existence of an insane delusion may in most cases entitle a prisoner to an acquittal, but it is quite possible to put cases in which it would not do so, and in which it ought not to do so. Men sometimes act consistently, as if their delusions were true. Such was the case of Mr. Greenwood (3 B. C. C. 444), who was a barrister, and chairman of quarter sessions. He disinherited his brother, under an insane delusion that he had attempted to poison him. Suppose Mr. G. had been a notoriously wicked person, and under the influence of the same delusion had murdered his brother, which of Dr. Ray's arguments would apply to his case? The man acts precisely as he probably would have acted had the facts been real. He may well associate them with their ordinary moral relations, for I suppose the case of a man habituated to crime. He may be conscious that he is violating the law, and may be careless whether he does so or not.

"I am I own at a loss for any argument in favour of exempting persons under insane delusions from punishment, except arguments to which the present arrangements of the law allow their full weight.

"It has been supposed that this view of the law was taken by Lord *Erskine* in *Hadfield's case*, and by the present Attorney-General in his hardly less celebrated defence of M'Naughten. I think that neither of these cases affords a real foundation for such an opinion. Lord *Erskine* said to the jury, 'You will have to decide whether you attribute it (Hadfield's crime) wholly to mischief and malice, or wholly to insanity, or to the one mixing itself with the other. If you find it attributable to mischief and malice *only*, let the man die. *If you consider it as conscious mischief and malice mixing itself with insanity, I leave him in the hands of the Court to say how he is to be dealt with. It is a question too difficult for me.*' It is a question, however, which must be dealt with, and I think that the illustration I have given shows that the manner in which those who would make every insane delusion a justification for crime propose to deal with it, is unsatisfactory.

"In Sir *A. Cockburn's* speech on the trial of M'Naughten—a speech which it would be presumptuous in me to praise—delusion was no doubt relied upon as proof of the prisoner's irresponsibility, but the argument was not that delusion *per se* and in all cases was a complete answer to a charge of murder, but that it was strong evidence to go to the jury of the incapacity of the prisoner to say whether the particular act in question were right or wrong. Man, argues Sir *A. Cockburn*, has intellectual and moral faculties—they act through the brain; when there is disease of the brain their action is disarranged; and he concludes, 'the mistake existing in ancient times, which the light of modern science has dispelled, lay in supposing that in order that a man should be mad—incapable of judging between right and wrong, or of exercising that self-control and dominion without which the knowledge of right and wrong would become vague and useless, it was necessary that he should exhibit those symptoms which would amount to total prostration of the intellect; whereas modern science has incontrovertibly established that any one of these intellectual and moral functions of the mind may be subject to separate disease, and thereby the man may be rendered the victim of the most fearful delusions, the slave of uncontrollable impulses, impelling, or rather compelling him to the commission of acts such as that which has given rise to the case now under your consideration.'

"The Attorney-General's position, in short, is that a man may be subject to delusions which, without depriving him of all his moral or intellectual powers, may prevent acts done under their influence from being wilful, or from being malicious. This no one doubts, but this is a very different position from the position that the presence of any insane delusion whatever will have that effect.

"Since many persons may be mad, and many persons may be subject to delusions, who would nevertheless be fitting subjects for the punishments of the law, it remains only to consider what is the nature of the operation of the existing law. How would it adapt itself to the various forms of madness which may be given in evidence under a plea of not guilty! I think that on fair consideration it will appear that it approves itself to common sense, and is entirely consistent with the general scheme and principles of legislation.

"The result of the law as laid down by the fifteen Judges in answer to the Questions of the House of Lords in 1843, illustrated by the authorities which I have cited, and by the practice of the Criminal Courts, is briefly this:—That certain states of mind are indispensable elements of crime. That the existence of any disease tending to rebut the legal presumption that such states of mind exist where certain acts have been done, may be given in evidence. That unless the evidence goes to the length of rebutting the presumption of those states of mind, it is not enough to entitle the party accused to an acquittal. In short, the inflexible rule of law is, that in every case, without exception, a wilful and malicious murderer (mad or sane) is to be punished.

"What constitutes malice is a question for the Judge: the existence of the facts from which the Judge is to infer malice is a question for the jury. Now the state of mind from which the Judge will infer malice is a state in which the prisoner is not prevented by any mental disease from knowing that the particular act in question is wrong.

"Some persons of great authority—amongst others Lord Brougham—have said that *wrong* means illegal. I think that if the Judges had meant illegal they would have said illegal; and I am confirmed in this opinion by the circumstance that most of them give a reason for using the word wrong in preference to the word illegal; which reason is, that, if the question were whether the prisoner knew that the act was illegal, the jury would be led to suppose that actual knowledge of the law was necessary, whereas the law presumes such knowledge conclusively. What, then, is the precise meaning of a man's being disabled by mental disease from knowing that a specific act is wrong?

"Each member of this sentence requires attention.

"1st. The person must be *disabled*. The law asserts that certain acts are wrong, and if any one chooses to act upon a different opinion he must take the consequence. Mere ignorance or mere difference of opinion with the legislator is no justification. Moreover, it is the ability which the law looks to, and not the actual knowledge. That is to say, the man must be in such a state of mind that it is his own fault if he does not steadily view, and pass a judgment agreeing with the judgment of the law, upon the quality of the act before him. A man may know that an act is right, or think that it is right, and yet be perfectly able to know that the law thinks it wrong; and if he is so circumstanced he is responsible to the law notwithstanding his private opinion, for the law tolerates no acts done in opposition to it, however honestly.

"He must be disabled by *mental disease*. It is not any disability that will be enough. A man may be disabled by passion or by prejudice from following the reasoning of the law; but unless it is mental disease which so disables him, either by weakening his intellectual powers generally, or by introducing into the circumstances of the case delusions of such a nature as to prevent his accurately judging whether the proposed act is wrong, he will not be excused.

"His disability must refer to the *specific act*. It must be observed that it is not required that the prisoner should be entirely destitute of all knowledge of right and wrong. This limitation was probably introduced by the Judges from the Scotch law. In Alison's* Principles of the Criminal Law, it is said, 'The great characteristic of insanity which originates in the general case, in an excessive turning of the mind to its own affairs, consists in an alienation of reason with reference to itself, and in the illusions under which it labours, and the chimeras it has nourished in regard to its own concerns. Few men are mad about others, or things in general,—many about themselves. Although, therefore, the pannel understands perfectly the distinction between right and wrong, yet, if he labours, as is generally the case, under an illusion and deception as to his own particular case, is thereby disabled from applying it correctly to his own conduct, he is in that state of mental alienation which renders him not criminally answerable for his actions.'

"He must be disabled from knowing that the act is *wrong*. It is upon this word that the greatest questions have arisen. I have before remarked upon the reasons why the difficulties which have been connected with it appear to me to be exaggerated. I will now attempt to show, that without any theory of morality at all, or in connexion with any such theory whatever, a very distinct sense may be attached to this word, and very distinct questions raised upon it for a jury. It must be observed, in the first place, that *wrong* means that which the law, and not that which the prisoner considers wrong. If it were not so, a man not believing in morality at all could not be protected by any amount of madness, for as he did not believe in wrong when he was sane, he could not be prevented from perceiving that an action was wrong by madness. Speaking with reference to any rule whatever, I think that in common language there would be a distinction between an irregular and a wrong act. The one violates the letter, and the other the spirit of the rule. If, in working a multiplication sum, a person were, instead of multiplying 7 by 7, to write 7 7's in a line and add them up, he would act irregularly; if he were to compute them to amount to 50, he would act wrongly.

"A somewhat analogous distinction, I think, obtains between what is merely illegal and what is wrong. A man may be said to act illegally who does some act which violates the letter of the law. If he acts illegally, knowing but disregarding the reasons which induced the Legislator to make the law, he does wrong.

"Thus, if a man had just a sufficient glimmer of reason left to remember as a fact that people were hanged for murder, but not enough to know the circumstances connected with murder which make it criminal, namely, the distress and insecurity which it causes, I think that he might well be said to be disabled by disease from knowing that murder was wrong. Upon any theory of

* "Page 465."

morality whatever, the circumstances which surround an act give that act its moral character, and the ability to distinguish enough of these to be able to appreciate the reasons of the law in forbidding the act, is surely a very different thing from the vestiges of memory which would suggest that the act was forbidden.

"Some acquaintance with the reasons of the law is presumed continually in its administration. If it were not so, the maxim that the law is the perfection of reason, and that what is no reason is no law, would be a mere boast, whereas, in fact, the greater part of the law of contracts and of wrongs—the law which regulates the common transactions of life—has grown up from the rational amplification of various elementary principles and rules. It may, therefore, be presumed that the criminal law is not a mere set of iron regulations punishing all who violate them, without regard to any other reason for doing so than the *sic volo sic jubeo* of irresponsible power, but a system laid down for the government of rational beings, whose responsibility depends upon their possession of such an amount of reason as may enable them to appreciate the grounds of some of those obvious enactments, without which no society ever existed.

"I think that the word *wrong* is thus understood by juries in general. If it were necessary to be more explicit, I should be inclined to think that the following would be very nearly equivalent to the ordinary question proposed to them,—Was the prisoner prevented by mental disease from appreciating the reasons for which the law has forbidden the crime of which he is accused, or from applying them to his own case?

"Applying the rule of law thus interpreted to the various cases which may arise, I think it will be approved of by common sense, and I much doubt whether any other would. To take the vexed questions of what are called moral and impulsive insanity: Can any course be suggested more reasonable than that of saying, Let these strange impulses be shown to be as they are often called *irresistible*, and they shall exempt the subject of them from punishment, because they sustain a traverse of the averment in the indictment that the act done was wilful?

"If the law is to rest satisfied with proof, not of an irresistible, but merely of an unresisted impulse, it gives a sanction to all sorts of crime, yet the person is as undeniably under the impulse of disease when he feels a resistible as when he feels an irresistible impulse. To illustrate this, I would refer to the case, which is still fresh in general recollection, of Mrs. Brough. Let us consider how far the public would have been satisfied if that case had been determined upon the ground that any person suffering under insanity was to be acquitted. I assume hypothetically that the facts of the case were these—as at any rate they well may have been,—That the woman, being unfaithful to her husband, thought of murdering her children from a sort of Medea-like fury, and that a rush of blood to the head acting on an excited brain was the immediate occasion of that thought being transmuted into action; in short, that if she had either been chaste or healthy the act would not have been done. As the law now stands, the question for the jury on this state of facts would be, whether, under all the circumstances of the case, the act was voluntary? If the law were altered, the question would have been, had disease anything whatever to do with the act? This mode of treating the case would have prevented the question as to whether the impulse was irresistible from being even raised. The law having declared that any insane impulse should be a justification, the only question would have been the existence of such an impulse. As it was, the verdict gave ground to many criticisms, even though it was in effect that the impulse was irresistible. Suppose the result of the trial had only been to show that there was such an impulse, what would have been the feeling on the subject?

"Apply the law as it stands to the case of insane delusions. Bellingham, I will suppose (for in all these cases I assume the facts merely for the sake of illustration), shot Mr. Perceval, because he was under an insane delusion that Mr. Windham had injured him, and under a sane delusion that the person at whom he had fired was Mr. Windham. A verdict of not guilty, under the law as it now stands, would have been equivalent to saying, the disease in Bellingham's mind, which produced the delusion, extended so far that he was incapable of understanding that the law would regard him as causing a public and private calamity, and as setting a bad example. A verdict of not guilty under the proposed amendment of the new law, would be consistent with a state of the prisoner's mind, affording as little excuse for what he did as his mistake as to the identity of his victim.

"Or take the case of Hadfield. Hadfield thought that he was our Lord; that it was necessary for the salvation of the world that he should die; that he ought not to kill himself; that firing at George III., or in his direction, he should be hanged, and that the world would be saved.

"Interpret *wrong* in this case to mean *illegal*, and Hadfield could not have been acquitted. The very reason of his conduct was, that his act was illegal, and that he should be punished for it; and yet if he were punished it is hard to say who ought to be acquitted. The verdict of not guilty actually returned amounted to this. True it is, that Hadfield knew what he was doing, and knew that he was breaking the law, but his delusions introduced into his mind a set of considerations—surrounded the act he was doing with a set of circumstances, which entirely prevented his estimating its character.

"Upon the facts which I have stated, I think no one will doubt that Bellingham was rightly punished (though, in fact, his trial was unjust, on account of the refusal of the Judge to postpone it for the collection of evidence), and that Hadfield was rightly acquitted. Could any course except that adopted by the law have secured that result?

"A suggestion, originating, according to Mr. Prichard, with the German jurist Hoffbauer, has been made, that the delusion should, for the purposes of justice, be considered as real; and that principle has to a certain extent been adopted by the fourteen Judges in their judgment referred to above. 'If a person,' ask the Lords, 'under an insane delusion as to existing facts, commits an offence in consequence thereof, is he thereby excused?' The Judges answer, 'assuming that he labours under such partial delusion only, and is not in other respects insane, we think he must be considered in the same situation as to responsibility as if the facts with respect to which the delusion exists were real.' It will be observed that the Judges do not say that the existence of an insane delusion, which, if real, would not justify the act done, may not be evidence to go to the jury on the question as to whether the prisoner knew that the act he was doing was wrong. Great injustice might be done to prisoners if this were not so.

"At the last Summer Assizes at Derby, a man was tried for murdering his child. It was proved that he believed that God had ordered him to do so; and he accordingly did it without concealment, but with every possible instance of contrivance and premeditation. Whether, if the delusion had been the fact, he would not have been still punishable *in foro humano*, is an extremely doubtful question. Persons with a settled sane conviction of such a doctrine—as was the case with the Thugs—were punished for what they did in consequence of it; but that such a delusion would or might introduce circumstances into the case, which would interfere with the estimate which the prisoner would form of the quality of the act he was doing, cannot be doubted, and a jury might very naturally say, a man under such a delusion could not be expected to understand that his action fell within the scope of the reasons which have induced the Legislature to forbid the murder.

"The law as it at present stands affords the insane every immunity consistent with the safety of society; for though mere proof of the existence of some insane delusion, or of what are called the moral and impulsive forms of insanity, will not *per se* justify a verdict of not guilty, yet it will in general raise such a presumption that the prisoner did not know that the particular act of which he was accused was wrong, that, in the absence of any proof of express malice, the jury will generally acquit upon that ground.

"In *Buranelli's case*—tried at the March Sessions at the Old Bailey—many instances of singularity of conduct on the part of the prisoner were given in evidence, and it was proved that he laboured under a positive delusion as to the symptoms of a particular complaint by which he was afflicted. There were, however, circumstances in the case which seemed to show that he had had a quarrel with the man whom he put to death. He was accordingly convicted and executed. If he had killed some one whom he had never seen before, and entirely without apparent motive, those circumstances, coupled with the strangeness of his conduct and the delusion of which he was the victim, would in all probability have procured his acquittal.

"I could hardly find an instance which illustrates more clearly the position for which I am contending; namely, that however the burden of proof may in the course of the investigation be shifted from one side to the other, the question to be ultimately solved is and ought to be, was the prisoner able to know that the act he was doing was wrong? The various misunderstandings which have taken place upon this subject mostly arise from confounding together the thing to be proved, and the means of proving it.

"No doubt proof of insanity generally, or of insane delusion specially, is strong presumptive evidence of the prisoner's irresponsibility; but it is evidence merely, and evidence of the effect of which the jury is the proper judge, and whatever faults may be found with juries, no one will charge them with giving too little weight to such evidence.

"I doubt whether it is possible to put a case of a person who wilfully and maliciously commits a crime whom the public would not wish to punish, if all the circumstances of the case were before them. I doubt whether any course of proceeding could make a nearer approach than is made by the present rules of law to the provision of means for the punishment of all such persons, and the exemption of all others.

"It is now an admitted principle of jurisprudence that the object of punishment is the prevention of crime, and that except in so far as it has that tendency it is an evil. The rules of law with respect to the punishment of madmen are in entire agreement with this principle, for it is notorious that mad people in general are as much acted upon by fear as those who are sane, as to those acts which are in any way under their own control. The cunning with which they will often conceal their insanity when examined on commissions of lunacy proves this conclusively.* The law as it now stands recognises in their case the distinction that it would be useless to punish them for acts of which they cannot appreciate the criminality, because for such acts they would not anticipate punishment. A man, for example, might abstain from murder because he might suppose that he would be hanged for it; but that would not induce him to abstain from breaking crockery, yet he might be under a delu-

* "When Martin, the incendiary of York Minster, was to be tried, various inmates of a madhouse were talking over his case. One of them remarked, 'Oh, they cannot punish him; he is one of us.' I have somewhere read of a madman who tried desperately to kill his keeper, and on being overpowered cried out, 'I will murder you yet; they can't hang me for it; I am mad!'

"In the debate in the House of Lords, on the 13th March, 1843, on this subject, Lord Brougham said, that Sir H. Halford had told him that madmen were as much, if not more liable to be influenced by fear than others."

sion that he was breaking crockery when, in fact, he was killing another man. So, too, it would be useless to punish a man for an act which though in fact wrong he could not recognise as such on account of delusion. If a man fancied that God had ordered him to put another person to death, the fear of punishment would deter him but slightly, if the delusion were strong, probably not at all; for the mere fear of pain or of death has less deterring influence over persons contemplating crime, than the fear of that universal and solemnly-pronounced disapproval of which an ignominious death is the outward and visible sign. In the case which I have supposed, this element of punishment would be quite wanting, for the man would say, 'If they knew the true quality of my action, they would approve of it. They put me to death not for what I have done, but for something else which they falsely suppose me to have done.'

"It has been ingeniously argued, that the punishment of the sane is enough to deter from crime both sane and insane, and that upon this ground madmen might be exempted from punishment.

"Nothing so entirely weakens the force of any course of conduct as capricious exceptions resting on no principle. Suppose it were enacted that out of 1000 persons convicted only 999 should be punished, and that every man on conviction should draw lots for the chance, would not the most ordinary knowledge of human nature tell us that such an enactment would diminish enormously the preventive effects of the law? The implied confession, that criminal law is only conventionally and traditionally necessary, that it is to be put into execution as reluctantly as possible, that crimes are not of much real importance, but that to satisfy existing prejudices they must be punished, would go far to destroy the moral force of any law whatever. I can see nothing but a less glaring form of this error in the proposal to free all madmen from responsibility; surely the proper course is not to snatch at an excuse for freeing a whole class from punishment, but to ascertain dispassionately the reasons which make it inexpedient to punish most of the members of that class, to extend impunity to those to whom those reasons apply, and to no others. This course the law at present adopts; can any other be suggested which would not involve it in gross injustice and inhumanity, or commit it to metaphysical and medical propositions of the truth of which it has no special means of judging?"

---

